# I Want to Become a Registered Nurse as a Non-Traditional, Returning, Evening, and Adult Student in a Community College: A Study of Career-Changing Nursing Students

**DOI:** 10.3390/ijerph17165652

**Published:** 2020-08-05

**Authors:** Luis Miguel Dos Santos

**Affiliations:** Woosong Language Institute, Woosong University, Daejeon, 34514, Korea; luismigueldossantos@yahoo.com; Tel.: +82-010-3066-7818

**Keywords:** associate degree, career changer, career development, community college, non-traditional, returning, evening, and adult students, nursing education, nurse shortage, registered nurse, second career, social cognitive career theory

## Abstract

The current significant human resource and workforce shortages of registered nurses (RNs) are impacting urban, suburban, and rural hospitals and healthcare facilities all over the globe, regardless of the entities’ economic and financial backgrounds. The purpose of this research study is to understand why non-traditional, returning, evening, and adult (NTREA) students decided to enrol at the Associate Degree in Nursing programme during their mid-adulthood? 40 s-career nursing students who are pursuing their nursing programme were invited to individual interview sessions and focus group activities on sharing and expressing the motivations in the New England region in the United States. Based on the theoretical framework of Social Cognitive Career Theory, the researcher concluded that family consideration and higher social status were two of the major themes. The study provided a blueprint for human resource professionals, health and social caring leaders, government agencies, policymakers, and researchers to reform their current nursing curriculum and health workforce policy to attract potential second-career nursing joining the nursing profession.

## 1. Introduction

The current significant human resource and workforce shortages of registered nurses (RNs) are impacting urban, suburban, and rural hospitals and healthcare facilities all over the globe, regardless of the entities’ economic and financial backgrounds [1]. In recent decades, the United States has invested a significant amount of financial aid and support both for secondary school graduates and for second-career changers and other non-traditional, returning, evening, and adult students (NTREAs) [2] who want to pursue career development in the field of nursing practice. Investments from the federal government, state governments, and county agencies have contributed to a potential increase in the number of non-traditional career pathways, and career development approaches in the nursing profession, as a large number of second-career individuals and career changers decide on nursing for their career development [3]. 

Second-career nursing professionals can bring to the medical field significant contributions in terms of industry experience and professional practices from their first careers [4], and much of that knowledge and those practices can be integrated into nursing management, administrative tasks, and interpersonal communication [5]. Therefore, the question facing the country is: In addition to fresh secondary school graduates, who will work in the health and medical care system? The answer to this question involves exploring new sources for nursing students and registered nurses and knowing the influences that motivate and encourage Americans to select nursing as a lifelong professional investment. This study focused on why non-traditional, returning, evening, and adult students [2] and second-career changers decide to choose nursing for their career pathway during the middle years of their adulthood. 

### 1.1. Brief Literature Review

The profession of nursing focuses on the issues of health, social care, counselling, and education [5,6]. The natures of the health, medical, and nursing professions usually do not have significant overlaps with business organisations, but increasingly, many Americans have decided to leave their original career pathways in business and other professions and to become registered nurses. Nursing education programmes and professional training for potential registered nurses have one of the fastest-growing enrolments in colleges and universities. Many nursing students have asserted that becoming health, medical, and social care providers allowed them to satisfy their goals and internal egos [7]. Because many adults had had extensive professional work experience in other fields before they entered nursing programmes, most understand the relationships and issues associated with a second-career change to nursing [8]. 

Although the United States is facing a significant nursing shortage, many associate degree programmes in nursing require interviews, exam scores, and placement exams due to a large number of applicants and interested students [9]. Because many of the existing nursing programmes do not have an open-enrolment policy, many non-traditional, returning, evening, and adult students and second-career changers cannot pass the programme admission requirements [2,10]. Privacy safeguards prevent most colleges and universities from publishing students’ background information, but it is not hard to imagine that secondary school graduates and youths with an extensive general education background and recent training for university entry exams, such as the Scholastic Aptitude Test and American College Testing exams, take most of the enrolment slots in nursing programmes. Therefore, due to the difficulties of changing to a second career and the issues associated with general education and university entrance exam requirements, the non-traditional, returning, evening, and adult students and second-career changers have lower enrolments in nursing programmes than traditional-age students do [2,10]. 

Second-career nurses are looking for opportunities to make a difference in people’s lives and thus are willing to be full-time students and to agree to sacrifice their immediate earnings for their educational voyage [8,11,12]. In addition to people with no prior college or university educational background, many individuals with at least a bachelor’s degree in a field other than nursing have decided to enter nursing education programmes because of their career motivations, interests, goals, and educational achievements [13]. Unlike the case with some professional medical specialists, such as physical therapists, it is not uncommon for other members of the medical public, such as general physicians and nurses, to have access to and observe in clinical environments. Thus, many understand that the health, medical, and nursing professions are positive and supportive of their life goals and potential lifelong career investment. Unsurprisingly, many second-career nurses advocate that nursing is a life-long investment and career development commitment rather than a job [3,14]. 

Another previous study [8] explored the transition to nursing practice experiences of the nursing students (i.e., first- and second-career changing nursing students) in the United States. The mixed research results (i.e., 15 qualitative data information and 122 quantitative data information) indicated that stressors and coping, the prevalence of burnout and presenteeism, and difficulty describing nursing’s role were three of their reasons and sources of stress and burnout as first- and second- career-changing nursing students. In fact, due to the changing working environment, many individuals experienced different levels of problems [15,16,17,18,19]. 

Topics on career development, and particularly on second-career development during mid-adulthood, have not yet received adequate research attention and exploration [20]. However, without a doubt, second-career nurses must be one of the most important sources for filling the significant gaps in the current health, social care, and nursing professions internationally. That is true, particularly for nursing professionals with extensive professional skills and experience in the industry. Due to the rapid development of society’s social, cultural, and economic backgrounds, mid-life career changes are not uncommon [21,22,23,24]. However, ensuring individuals’ career and financial security after they complete a nursing programme is vital because most second-career people have family and financial responsibilities. Because of the demand for health, social care, and nursing professionals continues to increase, studies about second-career nurses are needed [25]. 

### 1.2. Purpose of the Study

The purpose of this study was to use a phenomenological analysis [26] to explore the motivations for and reasons underlying the second-career changes by people in mid-adulthood who had decided to enrol in an Associate Degree in Nursing programme at a community college in the New England region in the United States. 

Because of the significant international human resource shortage of registered nurses, the results of this study will contribute to the field and should provide a blueprint for human resource professionals, healthcare administrators, university department heads, policymakers, educational researchers, nursing professionals, and government agencies in their efforts to reform and polish their current planning [9]. 

Encouraging people to change their career to a new one (i.e., in the health, social care, and nursing professions) is always a potential solution for filling the human resource gaps [27]. Therefore, expanding the understanding of career decisions and career beliefs always helps guide the management of educational and training programmes. Although some relevant quantitative information and data for different backgrounds and geographic regions already existed, this study will add important contemporary information about the backgrounds and interests of a unique group of participants undergoing mid-adulthood career changes to nursing. In short, one research question guided this research study, which was,

(1) Why did NTREA students decide to enroll in the Associate Degree in Nursing programme during their mid-adulthood?

Based on the abovementioned elements and backgrounds, based on the lens of the theoretical framework (i.e., the Social Cognitive Career Theory), and the perspectives from the participants, the outcomes of this study tend to provide recommendations about human resources and workforce management in the field of health, social care and nursing profession. Nursing school leaders, government leaders, policymakers, human resource planners, and scholars to reform and polish the current education and human resource planning for the next decades. Second, many of the current colleges and universities academic programmes and courses are designed for full-time and day-time students with long-term commitments. However, NTREA students usually cannot enrol at any of the full-time programmes due to various responsibilities. Colleges and universities leaders should take this opportunity to reform their part-time and evening programmes for NTREA students. 

### 1.3. Theoretical Framework

**Figure 1:**
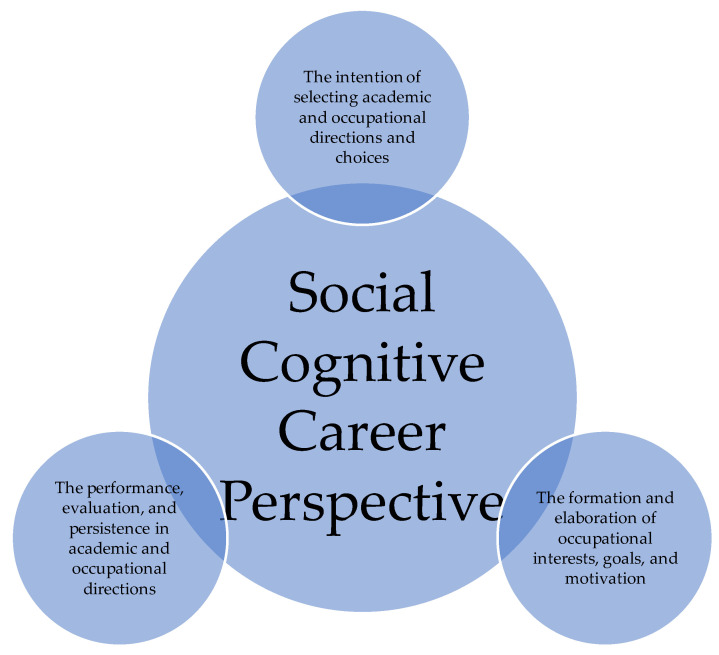
The relationships of the elements of the Social Cognitive Career Theory.

The current research study was guided by the Social Cognitive Career Theory (SCCT) [13,21,23,28,29,30]. The SCCT was developed based on the recommendations and elements from the ideas of Social Cognitive Theory by Bandura [31,32,33,34,35]. According to the guidelines of SCCT, the researchers advocated a learning model and cognitive behaviours [36] prompting people to understand, make sense, and establish their ideas. Figure 1 outlines the relationships. Three key points have been outlined, as the following,

(1) The formation and elaboration of occupational interests, goals, and motivation;
(2) The intention of selecting academic and occupational directions and choices;
(3) The performance, evaluation, and persistence in academic and occupational directions and intentions.

The SCCT highlighted the impacts and relationship between cultural, social, and economic elements and influences on people’s self-knowledge, self-understanding, sense-making process, and opportunity outcomes and results [16,37,38,39]. The process and element can be interconnected. For example, individuals’ career decision can be influenced by one or more than one element. To illustrate for this research study, it is sometimes observed that NTREA [2] nursing students decided to study a college degree and professional licensure programmes due to financial consideration. However, as social impacts may influence individuals’ financial incomes and consideration, individuals may change their mind during their educational voyage. 

As a result, based on the guidelines of SCCT [13], the researcher would like to use this theory to understand why NTREA students during their mid-adulthood decided to study their Associate Degree in Nursing in the New England region in the United States.

## 2. Materials and Methods

The researcher employed the qualitative research method [40,41,42] as the structure of this study. Unlike the quantitative research studies with surveys and questionnaires, qualitative researchers tend to understand the sharing, lived stories, personal background, feedback, and sense-making process of the individuals [43]. It is worth noting that qualitative data information is rich and in-depth as qualitative researchers need to access the participants and collect some *Why* and *How* [41] interview questions about participants’ background. Such studies usually gather in-depth background and sharing for the research question. 

### 2.1. Research Location

**Figure 2:**
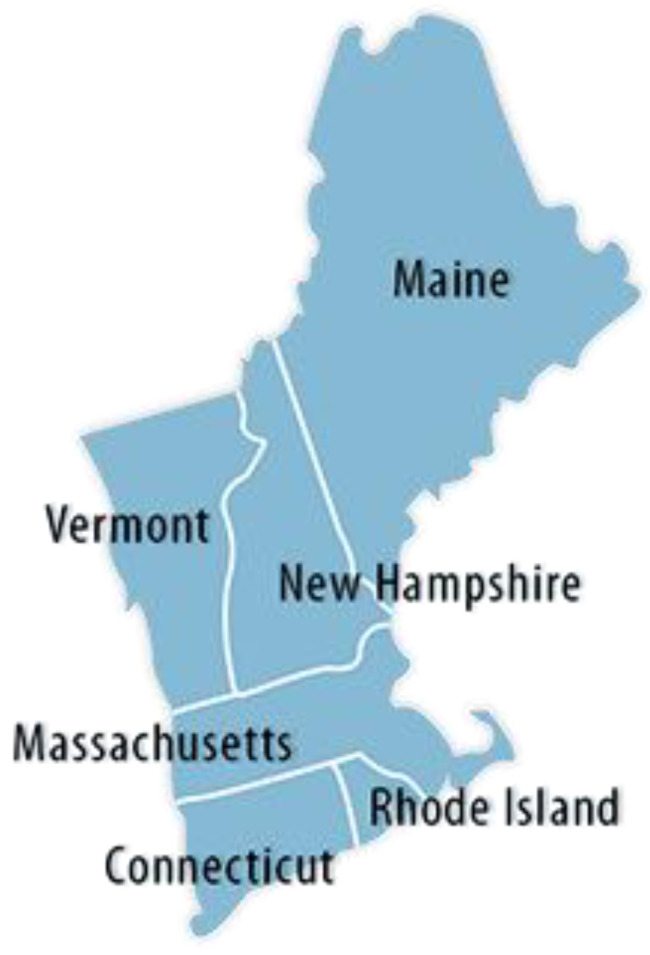
The New England region.

The New England region in the United States was the research background of this study (i.e., Maine, New Hampshire, Vermont, Massachusetts, Rhode Island, and Connecticut). First, unlike other small nations and countries, the United States is one of the largest nations internationally. Therefore, different groups and populations may have a different understanding, concepts, and values. As a qualitative research study, the researcher needed to select a targeted region for the research activity. In order to understand the holistic situations and issues of an American region, the researcher invited participants from each of the State within the New England region in the Northeast. Figure 2 showed the map of the New England Region. 

### 2.2. Participants and Recruitment

Forty (40) nursing students who are currently enrolled at an Associate Degree in Nursing were invited. All agreed to participate in this study. All the participants were NTREA students [2] and second career changers with previous working experiences after secondary school graduation. In other words, these participants were not traditional-age students who completed their secondary school qualification directly from secondary schools. All were American citizens or permanent residents and residents of one of the six states in the New England region in the United States. The researcher invited these participants with the purposive sampling strategy based on the personal network and connection. Please see Appendix A for the participants’ demography. The participants needed to meet the following criteria, which were:

(1) Never completed a postsecondary qualification;
(2) Never work in the nursing profession;
(3) Left school for at least ten years before this nursing programme;
(4) Be a resident of one of the six states in the New England region for at least two years.

The researcher, as an experienced education, public health, nursing, social caring, and psychology researcher in the North American and the Asian-Pacific regions, the researcher has established some networks within the fields. With the purposive sampling strategy, the researcher invited these participants based on personal network and referral from others. In order to invite these participants into the study, the researcher sent each potential participant an email invitation with the interview questions, nature of the study, risks, and the content form or agreement. As a result, 40 participants agreed on participation. After all of the participants agreed on the study, the researcher started to arrange the interactions and data collection procedure. 

### 2.3. Data Collection

**Figure 3:**
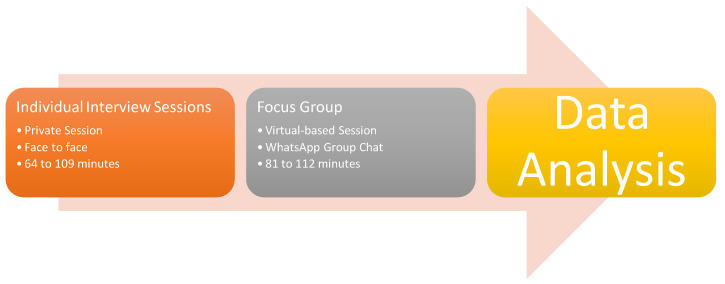
The procedure of the data collection.

Two types of tools were employed, including individual interview sections and focus group activities [44]. Please see Appendix B for the individual interview sessions questions, and Appendix C for the focus group questions. The individual interviews were conducted to explore participants’ understanding, lived stories, in-depth sharing, and sense-making process about their learning experience and career decision under a private sharing environment. The focus group activities were conducted to explore some similar background, social and cultural understanding, personal sharing, expectations, goals, interests, educational understanding, achievements, difficulties, and interests. Both research tools were useful in regard to listening to the voices and sharing by engaging the participants individually and collectively. As for the procedure of the data collection, please refer the Figure 3. 

All participants have the rights to participate or withdraw from the study at any time of the process. The general inductive approach was employed for the qualitative data collection and data analysis process. In order to seek useful data information for the research question, first, the researcher developed a set of interview protocol and interview questions. According to Seidman [45], privacy and confidentiality are important for lived stories sharing. Therefore, each individual interview section was hosted in a private room at public libraries, college classrooms, and community centres. No additional and third parties involved. Each interview lasted from 64 min to 109 min. 

Second, after each participant completed the individual interview sections, all participants were invited into eight focus groups activities via social media (i.e., WhatsApp Group Chat). Due to the location (i.e., six states within the New England region), not all participants could come to a targeted location for the focus group activities. Therefore, the online-based focus group activities overcame the issue. Five participants were invited to each focus group activity. In total, eight focus group activities were hosted. Each focus group activity lasted from 81 min to 112 min. 

After the researcher analysed the data information, the information was sent to the related participant for the member checking process. Due to the location issue, the researcher sent the related written transcripts to each participant via email. In addition, the researcher also invited each for a member checking interview for the purpose of confirmation. As for this member checking interview session via social media, the interview session lasted from 20 to 34 min. As a result, all participants agreed to their data information and approved their parts. 

### 2.4. Data Analysis 

The researcher first transcribed all the voice records and messages into written transcripts. After the researcher transcribed the oral and verbal data information from voices messages to written transcripts, (i.e., 40 interview sessions and eight sessions of focus group activities), the researcher collected more than 500 pages of written transcripts filled with rich, in-depth life stories and sharing from 40 s-career nursing students who were currently enrolled in one of the Associate Degree in Nursing programmes in the New England region in the United States. Based on the written transcripts, the researcher re-read the data information multiple times in order to figure out the connections and ideas between the participants. It is worth noting that the researcher merged the interview sessions and focus group activities sharing for each participant. Therefore, no particular categories for interview sessions and focus group activities. 

Some qualitative researchers [2,21,23,25,27,46,47,48,49,50] advocated that large-size data information should be narrowed down to meaningful themes and subthemes. Therefore, the researcher employed the general inductive approach [51] for data analysis. 

First, based on the recommendations from the general inductive approach [51], the researcher employed the open-coding technique for the first-level themes categories [41]. After the open-coding technique, the researcher categorised 28 themes and 32 subthemes. However, based on the recommendations, the themes and subthemes should be further narrowed down. Open-coding technique [41,42,52] is one of the useful tools for qualitative researchers to narrow down large-size data information into first-level themes and subthemes. Unlike quantitative research studies with statistics, the key to qualitative research studies is to narrow down the data information into meaningful themes. Therefore, the open-coding technique was appropriate. 

**Figure 4:**
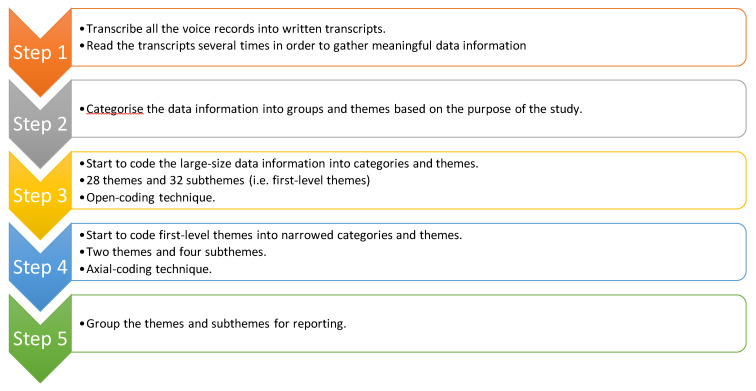
The procedure of the data analysis.

Second, however, after the first-level themes and subthemes were merged. The numbers of themes and subthemes (i.e., first-level themes) were large for reporting. In other words, it is not appropriate to report the findings with more than ten themes and ten subthemes. Therefore, in order to meet the standardised requirement for a qualitative research study, the researcher continued to employ the axial-coding technique for the second-level themes categories [17,21,22,37,39]. After the axial-coding technique, the researcher merged two themes and four subthemes for reporting [41]. For the data analysis procedure, please refer to Figure 4. 

### 2.5. Human Subject Protection

All signed and unsigned content forms and agreements, personal contacts, audio-recording, written transcripts, computer, and related materials were locked in a password-protected cabinet. The researcher was the only person for any access. After the study was completed, the researcher immediately destroyed and deleted all the related materials for personal privacy. Due to the content forms and agreements between the researcher and the participants, the college, detailed address, age, skin colour, and race information would not be outlined. Each would be given a pseudonym [41]. 

All subjects gave their informed consent for inclusion before they participated in the study. The study was conducted in accordance with the Declaration of Helsinki, and the protocol was approved by the University Ethics Committee (2019–2020/Fall/Winter). 

## 3. Results

Based on the research question of this study, the researcher categorised the data information into the following themes and subthemes for reporting. As a study for the nursing education and nursing workforce management, the researcher aims to understand why did NTREA students decide to enrol at the Associate Degree in Nursing programme during their mid-adulthood. In fact, a second career-changing decision during the mid-adulthood is not an easy step, particularly for individuals joining the vocational-oriented programme (i.e., nursing) which required a large part of internship and placement. 

Through this qualitative study, the researcher was able to conduct an inductive analysis of the data and establish themes and subthemes, answer the research question, and discuss the findings. Analysis of the data information yielded two themes (family considerations and personal goals/improved social status) and four subthemes. The findings discussed below are supported by verbal quotations from the transcripts. Table 1 refers to the themes and subthemes of this study. 

### 3.1. Family Considerations

In contrast to fresh secondary school students, university graduates, and youths without family and financial considerations, most of this study’s participants had family responsibilities as parents and/or caregivers of their own parents [53]. Therefore, more than half of their sharing and life stories concerned how the career change to nursing might provide different positive outcomes for their family members. All participants expressed that their strongest intention was based on their goals for their family members and not on their own educational achievement and interests [13]. Although a few indicated that they wanted to join the nursing profession because of their childhood experience, most highlighted that their goals for their family, financial resources, work schedule, and positive influences on their children were their priority. The following sections elaborate on the participants’ rich and in-depth sharing and subthemes. 

#### 3.1.1. Better Financial Resources

The four subthemes included quoted sharing and opinions from all participants. In other words, all participants shared similar subtheme categories. The subthemes *money* and *financial resources* were shared more than 120 times in both interviews and focus group sessions. Therefore, second-career nursing students tended to be influenced by financial resources as their primary goal [13,23]. From their perspective as parents, all desired to make more money for their school-age children––for example, one believed that the salary from her nursing position could provide better education for her children, saying:

I want my children to have a better education and life…after-school programmes with piano playing and sports cost money…working as a housewife doesn’t help…I needed to make extra earning[s] to support my kids…my family cannot just rely on my husband’s [re]sources as all family [desires]and programme fees are not cheap at all…

(P#15, Housewife)

Another participant also shared a similar idea, saying:

Although I agreed on the idea about man should be the one who makes money for the family, I still want to take some responsibilities for money-making…I want to offer a better future for my children…although YMCA offers some cheap courses or after-school programmes, we still need money and tuition fees for those courses…I need to be a useful person to make money for my family…

(P#17, Housewife)

Although many indicated that the government did not require such after-school activities, all wished to provide a meaningful childhood to their kids. The researcher captured two participants sharing, said:

…I lived in a poor urban community when I was young…I couldn’t experience any after-school sports life and activities due to my family’s financial problems…I wanted to learn piano but I couldn’t as my father did not have much extra money…I cannot allow this happen to my kids again 40 years later.

(P#2, Supermarket Associate)

Poor is not illegal…but without money, we cannot do anything…I wish my children can be useful people for our community and country…so I need to make money to support my children for additional learning…or life experiences…even reading clubs and Sunday schools…we still need to pay a fee for the community supporting fee…so I need to give them a meaningful childhood…

(P#18, Housewife)

Based on the sharing, many indicated that financial considerations and resources took important roles in their decision-making process. For example, more than three-fourths indicated that they would need to live with food stamps if they could not upgrade their skills, saying:

The economy is very hard for high school graduates and unskilled people, like me…both of us [husband] are working as labour workers with the minimum wage…this is not a way to continue…at least I don’t want [my]children to [repeat]this … I need to go to school and be a useful person…this nursing programme [has] allowed me to upgrade myself…

(P#1, Fast Food Restaurant Server)

In addition to the participants with parental responsibilities, several single participants indicated that they sought a stable financial income and earning support for their living standard and spending needs for their parents [54,55,56,57]. Although the single participants did not have significant spending for their children, many still needed financial resources for food, rental fees, and other costs for their aged parents. People of colour and minorities mostly shared such expressions. Many indicated that working for a basic income could not satisfy their needs as a person in society, saying:

I am not smart, but I can still work…I can work for many labour jobs…I want to seek a position…I spend a certain amount of time and receive the balance amount of money and salary. As I am working in [in-home] social caregiving, I want to work as a nurse for a better salary with similar responsibilities

(P#26, Social Caring Provider)

It is true that without enough money, but just for my basic needs…I cannot be promoted or gained my personal enhancements…I want to improve myself as a useful person in the community…And I think being a nurse is a good way to go…so, after I asked my families recommendations…I want to study the nursing programme for a better life or change

(P#33, Housewife)

In short, many participants, particularly parents, had decided to go back to school, with their children and the financial health of their family as one of their strongest motivations for studying nursing for their career development. 

#### 3.1.2. Better Work Schedule Management 

“*I can take the overnight shift schedule, as I can spend the day-time with my children*” (P#30, Supermarket Associate). It is worth noting that the idea of an “on-shift schedule” was mentioned 123 times, according to the study’s transcripts. 

Single and married participants had different types of responsibilities, from family work to personal development, that influenced their decision-making processes and career development endeavours during their transition to nursing during their mid-adulthood. Because of the social bias about traditional working hours (i.e., from 9 AM to 5 PM), the general public often neglects to appreciate the difficulties and flexibilities of working professionals who take the on-shift duties. Nursing and medical professionals, for example, usually need to work for the whole year without a holiday. However, the participants of this study asserted that they wanted to take an on-shift working schedule so they could manage their daily activities with their children and others [1]. For example, a participant reported that the on-shift schedule allowed him to do some banking activities and governmental work during regular office hours, saying:

The traditional office hour positions do not allow me to go to the bank and government works…also, I cannot even go to interviews due to the schedule conflicts…the on-shift schedule allows me to manage my plan and do something I need to do besides jobs…

(P#39, Bank Associate)

Many parents indicated that they could spend much more time with their children if they took the overnight shift at the hospital. One shared an expectation about time management, saying:

I can work at night once my sons [go to] sleep at night at 9 PM…I work at 11 PM in the hospital for the night shift…my husband takes care of my sons’ morning tasks, and I take care of the after-school activities. We can make sure there is an adult at home…

(P#36, Hotel Associate)

Another parent also shared a similar experience in favour of the children’s advantages and the time arrangement, saying:

I can send my children to the school on my way to the hospital…if I cannot do that, I can send them to the school bus if I work for the morning shift…I will be home by 4 PM and they finish their after-school programmes at 3:45 PM…[the] overnight shift could be easier, but at least I can work on it…

(P#37, Hotel Associate)

In particular, most parents indicated that the overnight shift allowed them to handle their family responsibilities during the day-time and to work at night. Although a previous study explored that some nursing professionals disliked the on-shift schedule due to its time management conflicts, many participants in this study expressed positive feedback [58]. As a result, they did not need to stay at home as a housewife with limited incomes and earnings. 

In addition to the advantages of a balanced schedule for family and children’s concerns, a large group also maintained that the traditional office hours were not suitable for their outcome expectations [13,59]. Many indicated that in addition to the office hours, many positions in the service management and hospitality industry required an on-shift schedule due to the industry’s non-stop business operations. Such operations and management also happen in most of the health and social care professions. One participant in this study said:

I enjoy the on-shift working timetable as we can enjoy different assignments and patients every day. Unlike my current job with the same tasks, [with the] different shift[s] at the hospital…morning, afternoon, and overnight…the tasks are different from day to day…and I also work with different co-workers and people…I like this type of working environment…

(P#19, Clerk)

In short, nursing is a challenging career that can require medical professionals to handle different patients and accidents daily, and the participants not only found that requirement to be satisfying, they also understood the operation and appreciated the on-shift scheduling. 

#### 3.1.3. Better Image for their Children and Family Members 

In addition to nursing’s financial considerations and scheduling management opportunities, many participants stated that the professional image and career development of nursing professionals increased their mental position with their children and family members. Without a doubt, family considerations were always placed as the priority of these groups of participants. One said:

If I d[id] not have my children, I w[ould] not come back to school. I c[a]me back to school because I have to provide a better future for my kids and my family…I want to be a positive model for my children to learn…from my hardworking image…I need to tell my kids your mom is strong…and you should be strong too…

(P#9, Housewife)

Questions about how the expectation of presenting positive images for their children and family members influenced their career decisions were asked in both the individual and focus-group activity sections, so all shared their opinions on this issue. Several significant opinions are listed below [60,61,62,63]. For example, a participant believed that her own positive modelling could be a learning experience for her son, saying:

I always believed modelling is a way of learning. I learned to be a strong woman from my mom. My mom worked very hard to take care of my sibling and my grandparents during her mid-age. I absorbed this idea from my mom…I need[ed] to be a strong mom after I married. My son is in high school now. I want to build up a strong image [for] my son…you[r] mom can be a very strong student and mom as well…

(P#24, Housewife)

Additional, similar sharing about becoming a positive image and learning model for one’s children were captured [60,61,62,63]. One participant believed that children should have a positive learning model during their young age in order to motivate their learning and development. That participant indicated that the parents should be such a learning model, saying:

Both of my parents used their hands to raise my sibling and I up when they were young. I used to work as a housewife for a decade…I think I am a bit lazy if I compare to my mom’s experience. I told my children that their grandma always worked from 9AM to 9PM many years ago…but I don’t work…although it is a different situation and social-economic system…I need to be a positive image and model [for]my children

(P#35, Housewife)

Both positive and negative images and behaviours always influenced the behaviours and development of children. In addition to the statements from parents with young children, three interesting sharing were captured from parents with 12^th^-grade students who were planning to go to university the next year. Those participants believed that their own modelling (i.e., going to community college as a student) could encourage their children’s mental development and social cognitive learning. For example, a participant shared that she intended to be a positive model at university for her children, saying:

…I go to college during mid-age…my children will go to college soon too…so we go to college at the same time. I want to encourage my children to…pursue their dream and seek a college degree…not like me…just a high school graduate for more than three decades

(P#4, Housewife)

Another similar life story about attending college with her children was shared by a third participant. Interestingly, that participant was enrolled at the same community college with her son that semester as classmates in the same general education course. Both encouraged each other and shared materials and group project ideas. The mother said:

I can study with my son…this is very rewarding…as a mom, I d[id]n’t know how can I enter the society and community of my children…[but] this college and education and degree [work] engage[s] us…we learn, we grow, and we are getting closer to each other

(P#13, Housewife)

In short, based on those findings, the researcher found that the ideas of modelling from both the SCCT [13,59] and Social Cognitive Theory [60,61,62,63] strongly influenced the career development and career decisions of these groups of study participants, because they tended to become learning models. To illustrate such benefits to both young children and traditional-age university students, the participants shared significant stories about their efforts to motivate their family members for a better future. With the supports of the previous literature and studies, the researcher could conclude the connections and relationships between the SCCT and the decide-making process of the participants. 

### 3.2. Higher Social Status for the Future 

Unlike fresh secondary school graduates and other youths, second-career nurses usually have already acquired decades of working and life experience before joining the nursing education programme at the community colleges. Therefore, they have numerous responsibilities, personal backgrounds, considerations, comparisons, and concerns, from education to social biases and discrimination [64,65,66]. Based on the sharing from the study’s participants, many indicated that seeking a college degree would help them to achieve a higher social status that they then could promote as valued skilled professionals for their communities and country [67]. 

Although the United States federal government and state governments sometimes provide financial support for unemployed, unskilled, and low-income workers, many such individuals have asserted that they need to use their hands and labour to seek adequate resources to support their family. Such modelling of hard work can encourage children to seek and achieve a better future [22,30,68,69,70]. For example, one participant told that she intended to become a skilled worker for her community because she wished to invest her energy back into her hometown, saying:

…many of my neighbours are college graduates, they always contribute their energy and spare time to the community…such as community centres, libraries, weekend reading groups and so on…but I am useless. Therefore, I need to be a skilled worker and contribute my energy back to my hometown…

(P#29, Cashier)

#### 3.2.1. Registered Nurse Positions as a Long-Term Career Promotion 

Many stated that they wanted to set their long-term career development pathway in the direction of health and social care professions, particularly the nursing profession, after completing their degree programme. For example, a participant admitted that during the previous decade she had spent almost all of her savings towards her nursing degree programme, saying:

I am spending all my savings as I have to work as a full-time student without [an] additional job commitment. I have to focus on my study…I cannot work anymore. But I think it is worth it. I want to become a nurse, and this is my lifelong investment…I believe no pain, no gain…

(P#8, Restaurant Server)

The statement “*long-term and lifelong investment*” was shared many times by the participants, with many advocating that their college degree could help them to achieve their goals as registered nurses in the future. For example, several participants shared that a college education was expensive but worth the investment. The knowledge, skills, abilities, and leadership gained from their group work, placements, vocational training, communication interactions, and professional knowledge were hard to obtain without the guidelines from a college education. The researcher captured two statements, stated:

…college is a place for us to train up our brain…nursing education, as a vocational training programme…allowed us to learn the skills from placement, professors, and experienced nurses at both [the] hospital and classroom environment. Even if I don’t work as a nurse or retire from the hospital in the future, I still gain some valuable skills and knowledge as a lifelong investment

(P#32, Janitor)

…I used to plan to do some community services and Sunday school volunteering…but I think I should train up myself…as a useful person in the community…Sunday school is good…but nursing education further allowed me to upgrade myself as a nurse or useful medical professionals…for my country…

(P#5, Housewife)

It is worth noting that although operational skills (e.g., office tasks) and home economics (e.g., housework) are vital in many societies, most people have the desire to further improve themselves by receiving additional skills from college. For example, the researcher captured an interesting sharing from a participant with nearly two decades of experience as a security guard at a hospital, who said:

I help the medical professionals to mark down the schedule…the ambulance operation…I help to move the patients…I guide the patients to different levels and floors…but I am the assistant as I am unskilled. I want to invest my soul, my skills, and my career development for the life-long changing. I believe working in a hospital is very meaningful to our community. I came to this nursing programme because I want to help my community for life-long investment…not only [for] me but my town

(P#21, Security Guard)

In conclusion, in accord with the guidelines of the SCCT [13,59], and the research question of this study, this study’s results demonstrate that financial considerations and personal goals play important roles in individuals’ decisions to switch their career pathway and commit to the nursing profession during mid-adulthood. The result of this study might reflect on two previous studies about how personal goals and financial consideration may influence their decision-making process. Although the participants were from different parts of the region and had various backgrounds and responsibilities, most believed that without a stable financial income, they could not continue their standard of living [21,39]. Also, specifically among parents, many maintained that gaining a favourable image (e.g., going back to school during mid-adulthood and becoming a registered nurse) could serve as a positive model for their family members. Such thinking echoed both the SCCT [13,59] and the Social Cognitive Theory’s [31,32,34,35,60,71] exposition and described how such elements or goals could impact individuals’ decision-making processes. 

## 4. Discussions

The current study investigated why did NTREA students decide to enrol at the Associate Degree in nursing Programme during their mid-adulthood. By employing the lens of the Social Cognitive Career Theory (SCCT) as the theoretical framework, the researcher categorised family consideration and higher social status for the future as two of the main themes, reasons and motivation for their decision. According to the SCCT [13], individuals’ career decisions can be influenced by their personal goals, interests, and educational achievements. In accord with that theme, all of this study’s participants indicated that they always put their family members and children as their priority (i.e., as their personal goals) [21,22,72,73]. Reflecting to a previous study [20] about second-career engineers, the career change decisions reported in this study were without a doubt made due to the participants’ family issues, with one saying, “*Many of my life decisions were made due to my family members, my spouse, my children, and my parents…mid-aged parents usually do not have many choices…I would save my choices to my children and family members*” (P#26, Housewife). With the reflection of a previous study, the results indicated that parents from low-income families tended to spend their resources on their children for after-school programmes, even if they had limited financial resources [74]. With the reflection of in this study, all parents echoed the findings of the earlier research and indicated that the additional earnings from a nursing position could increase the educational level and life standard of their children and allow their children to access additional achievements and learning by, for example, after-school programmes.

Although the study’s participants were raised in different families and various geographic areas in the New England region, similar opinions about family responsibilities and the connections to financial resources ran through their reports. The ideas might reflect on a previous study. The results indicated that financial considerations could play significant positions and roles for individuals in their choices for their career development and their educational direction [75]. For example, other studies have explored the relationship between job loss and employment, based on the SCCT [13,21]. In this study, the participants echoed the previous studies and expressed that their family needed additional resources for housing mortgage and bills and that their previous careers and positions couldn’t support them. Previous studies [21,22,23,29,30] indicated that the expectation of an outcome plays an important role in how individuals select their career development and how individuals undergo their decision-making processes. In this study, most participants believed that the promise of additional financial resources informed their motivation and decision-making processes for transitioning to nursing from their previous career pathway. The participants’ behaviours reflected and echoed the SCCT [59,69,76,77] factors and how individuals should be treated.

In terms of working schedule management, many indicated that the nursing working schedule allowed them to manage their family responsibilities as parent and professional staff. With the reflection of a previous study [58], the sharing from the participants echoed the ideas about working schedule and working hours are one of their biggest concerns. Most indicated that keeping non-traditional office hours and schedules allowed them to manage their daily activities around their children’s responsibilities [21]. Many indicated that in addition to the office hours, many positions in the service management and hospitality industry required an on-shift schedule due to the industry’s non-stop business operations. Such operations and management also happen in most of the health and social care professions. A previous study [3] showed that nursing faculty members liked to take on unusual responsibilities, and indeed nursing and medical professions always provide new challenges and tasks with their daily operations, as one said, “*I don’t want to work as an office worker, nursing professional…fit my personal schedule and family responsibilities…*” (P#9, Housewife). With the reflection of many previous studies [21,37,70], almost all shared their appreciation that the schedule flexibilities would allow them to expand their interests in their potential nursing positions for both family and personal reasons. Those revelations reflected the SCCT [13,69,76,77] elements about outcome expectations for personal satisfaction in light of the workplace challenges and flexibilities of their daily tasks and activities.

In the sharing about the model of their children and family members, a previous study indicated that many nurses tended to establish a positive image and model [60,61,62,63] as learning tools [78]. Indeed, only a few of this study’s participants were single people without children. Many considered that their career development and activities should be based on the development of their children and planning of family members. With the reflection of a previous study, family members and parents’ images and behaviours always influenced the behaviours and developments of children [79]. Furthermore, the image of strong and hardworking parents did not influence just a single participant’s decision, but the choices of a large number of people.

In terms of a nursing career as their long-term career promotion and development, almost all participants indicated that career promotion is one of their significant considerations. Many previous studies have indicated that often, second-career nursing students in their mid-adulthood find it difficult to rearrange their time management, spend their financial savings, and balance their responsibilities because family and financial issues are vital, particularly for parents with additional responsibilities [8,20,22,80]. Therefore, enrolling in a nursing education programme must be planned and discussed for a variety of reasons and purposes. The results from this study echoed most of those previous studies, in that most participants maintained that their achievements in their nursing programme allowed them to register and become a registered nurse, which is one of the most important elements. With the reflection of a previous study, the results of this study indicated that many participants had the desire to become skilled professionals and receive the appropriate training [81] to gain both general skills (e.g., communication, interpersonal behaviours, foreign language, time management, and mathematics) and specific skills (e.g., social caring, patient management, bookkeeping, data collection and analysis, and hospital management) from their college degree programme in order to upgrade themselves as skilled professionals [82].

## 5. Limitations and Future Research Direction 

Although this research study provided the linkage between the SCCT and the behaviours of mid-aged NTREA nursing students in the New England region in the United States, it has several limitations. First, a qualitative research methodology [41] with the application of the general inductive approach [51] can be limited to be collecting data from different individuals. 

Second, due to the limited numbers of participants, the current research study could only invite mid-aged 40 NTREA nursing students. Future research can expand the population to a larger-size research study in order to cover a wider understanding. 

Third, due to the size of the United States, the researcher could only target the New England region as the mean. However, the understanding, feedback and career perspectives of people, groups, individuals, and populations in different parts of the United States could be different. Therefore, future research studies can cover different parts of the nation for wider coverage. 

Fourth, the current research study only covered American citizens and permanent residents in the United States. However, as the United States always welcomed international students for different types of academic and vocational programmes, international students’ voices may be covered for future research studies. 

Fifth, due to the nature of this study, this study tended to focus on the career decision and career perspective of a small group of mid-aged NTREA nursing students in the New England region. However, the experiences, sense of belonging, and learning strategies are not examined. Therefore, future researchers should seek understanding, feedback, and perspectives about these elements in order to expand the overall performances and holistic pictures for a better environment. 

Sixth, the current study tended to focus on the problems and reasons why NTREA students decided to enrol the nursing education programme at one of the community colleges in the United States. Future research studies should expand the scopes to the barriers and problems they encounter during the programme and after graduation. 

## 6. Conclusions

This research study has contributed to research practices devoted to nursing education, career perspective and human resource trends in two directions. First, the shortages of health, medical, nursing, social caring, and counselling professionals can be a critical and significant element internationally. This research study and its related outcomes served as the blueprint for nursing school leaders, government leaders, policymakers, human resource planners, and scholars to reform and polish the current education and human resource planning for the next decades. 

Second, many of the current colleges and universities academic programmes and courses are designed for full-time and day-time students with long-term commitments. However, NTREA students usually cannot enrol at any of the full-time programmes due to various responsibilities. Colleges and universities leaders should take this opportunity to reform their part-time and evening programmes for NTREA students. 

## Figures and Tables

**Table 1:** Themes and subthemes.

Themes and Subthemes		
3.1.		Family Considerations
	3.1.1.	Better Financial Resources
	3.1.2.	Better Work Schedule Management
	3.1.3.	Better Image for their Children and Family Members
3.2.		**Higher Social Status for the Future**
	3.2.1.	Registered Nurse Positions as a Long-Term Career Promotion

## Appendix A

**Table A1:** Participants’ Demography.

Name	Occupation	State
Participant #1	Fast Food Restaurant Server	Maine
Participant #2	Supermarket Associate	Maine
Participant #3	Housewife	Maine
Participant #4	Housewife	Maine
Participant #5	Housewife	Maine
Participant #6	Housewife	Maine
Participant #7	Housewife	Vermont
Participant #8	Restaurant Server	Vermont
Participant #9	Housewife	Vermont
Participant #10	Housewife	Vermont
Participant #11	Housewife	Vermont
Participant #12	Sales Associate	Connecticut
Participant #13	Housewife	Connecticut
Participant #14	Sales Associate	Connecticut
Participant #15	Housewife	Connecticut
Participant #16	Sales Associate	Connecticut
Participant #17	Housewife	Connecticut
Participant #18	Housewife	Connecticut
Participant #19	Clerk	Massachusetts
Participant #20	Sales Associate	Massachusetts
Participant #21	Security Guard	Massachusetts
Participant #22	Housewife	Massachusetts
Participant #23	Housewife	Massachusetts
Participant #24	Housewife	Massachusetts
Participant #25	Housewife	Massachusetts
Participant #26	Social Caring Provider	Massachusetts
Participant #27	Housewife	Massachusetts
Participant #28	Housewife	Massachusetts
Participant #29	Cashier	Rhode Island
Participant #30	Supermarket Associate	Rhode Island
Participant #31	Sales Associate	Rhode Island
Participant #32	Janitor	Rhode Island
Participant #33	Housewife	Rhode Island
Participant #34	Housewife	Rhode Island
Participant #35	Housewife	New Hampshire
Participant #36	Hotel Associate	New Hampshire
Participant #37	Hotel Associate	New Hampshire
Participant #38	Sales Associate	New Hampshire
Participant #39	Bank Associate	New Hampshire
Participant #40	Housewife	New Hampshire

## Appendix B

Individual Interview Session Questions

(1) Why do you want to study the nursing programme at a community college?
(2) What made you want to exercise this career switching from current position to potential registered nurse? Why and How?
(3) Why would a community college become a tool for your education? Why not other alternative routes?
(4) How would you describe your motivations and reasons for this nursing programme?
(5) For the enrolment of this nursing programme, have you ever considered nursing as your career pathways? If so, may you please tell me more?
(6) As a non-traditional, returning, evening and adult (NTREA) nursing student, how would this status change and describe your educational experience?
(7) Would the NTREA student status positively or negatively impact and influence your educational experience, educational decision, career decision, and personal experience?
(8) Would your occupational interests, goals and motivations positively or negatively influence your educational experience, educational decision, career decision, and personal experience as NTREA nursing student?
(9) Any follow-up questions.

## Appendix C

Focus Group Questions

(1) As NTREA nursing student at a community college, how would everyone describe your experiences? Please share, and we can see there are there any core similarities and differences.
(2) What are the main motivations and reasons for everyone to join the nursing programme and the nursing profession after the degree? May you please describe your understanding?
(3) Would your personal goals, educational background and previous experiences influence your decision?
(4) Do you think will this nursing programme help you to achieve your personal goals and expectations? Why and how?
(5) Do you think would your background as NTREA students positively or negatively influence any of your experiences? Why and how? For any directions.
(6) Any follow-up questions.
